# Influence of vanadium oxide on the structural, optical, mechanical and dielectric properties of cadmium borate glasses

**DOI:** 10.1038/s41598-025-09064-1

**Published:** 2025-07-28

**Authors:** A. Kh. Helmy, Takwa E. Ellakwa, Gehan T. El-BassyounI, M. A. Azooz, M. A. Ouis, Mohammed A. Taha

**Affiliations:** 1 Glass Research Department, National Research Centre (NRC), Giza, 12622 Egypt; 2 Physical Chemistry, Faculty of Pharmacy, Egyptian Russian University, Cairo, Egypt; 3 Refractories, Ceramics and Building Materials Department, National Research Centre (NRC), Giza, 12622 Egypt; 4 Solid State Physics Department, National Research Centre, Giza, 12622 Egypt; 5 Pharos University in Alexandria, Canal Mahmoudiah Street, Smouha, Alexandria, Egypt

**Keywords:** Cadmium borate glasses, Mechanical properties, Electrical properties, Dielectric properties, Optical properties, Materials science, Materials for optics

## Abstract

The melt-quenching method has been employed to fabricate 30B_2_O_3_–(70−x)CdO-xV_2_O_5_; x = 0, 1, 2, 3, and 5 mol% glasses. The physical, optical, mechanical, and dielectric properties of the prepared glasses were measured. Structural FTIR spectra and their related deconvolution process showed distinct absorption bands, from which some parameters especially, the N_4_ ratio, could be determined by calculating the area under peaks. The successive addition of V_2_O_5_ up to 2 mol% at the expense of CdO in the glass network led to an increase in density from 4.27 to 4.69 g/cm^3^ for the prepared glasses while adding more V_2_O_5_ led to a decrease in density. The molar volume showed the same trend that occurred in the density, but in an opposite direction, as it decreased by increasing V_2_O_5_ content till the 2 mol% samples, then increased. The optical energy gap values (Eg) were calculated and were found to decrease from 2.95 to 1.35 eV with increasing levels of V_2_O_5_. With varying V_2_O_5_ concentrations, the mechanical characteristics of cadmium borate glasses increased up to 2%, declined at 3%, and then improved once again at mol%. The maximum microhardness, fracture toughness, and Young’s modulus were 624.12 HV, 2.87 MPa.m^0.5^, and 63.71 GPa for samples contained 2 mol% V_2_O_5_ which improved about 14.91, 32.26, and 21.84% compared to unreinforced glass sample. In addition, adding V_2_O_5_ and increasing the frequency had a positive effect on the electrical conductivity with the real and imaginary dielectric constant, and tan δ is the opposite. The electrical conductivity and tan δ of glass containing high V_2_O_5_ at a frequency of 20 MHz were 1.45 × 10⁻^2^ S/cm and 0.0619, respectively, at a frequency of 20 mΩ.

## Introduction

Glass has grown to be a significant and essential component in a variety of industries, including communications, photovoltaics, optoelectronic devices, automobiles, and building materials^1–4^. In light of the growing need for materials with novel qualities, particularly in the field of optical devices, glasses have recently attracted the interest of researchers^5–7^. Among other glasses, borate glasses are the most widely used network former because of their superior optical, electrical, thermal, and chemical durability^8–11^. Borate (B_2_O_3_) glass is a highly effective glass forming that has a low melting point, great transparency, high thermal stability, and strong solubility of rare earth ions. Compared to silicate glasses, the alkali ion activity in borate glasses is more convoluted.

B_2_O_3_ glasses are intriguing materials for a range of cutting-edge applications. Originally made up of random boroxol rings connected by B-O-B bonds, the melting process helps to form the structural units [BO_3_] and [BO_4_] in borate glass. Depending on the type and concentration of the modifying cation in borate glasses, these units can change from BO_3_ to BO_4_ units and vice-versa. This conversion is essential for altering the characteristics of glass material because alkali oxides are known to be potent modifiers that can alter the coordination of boron atoms, depending on the alkali oxide content ratio^12^. When heavy metal oxides like bismuth oxide (Bi_2_O_3_), lead oxide (PbO), and cadmium oxide (CdO) are doped into borate-based glasses as network modifiers, attractive characteristics may result. The scientific community has taken notice of CdO due to its function in improving glass properties. The high ability to form, high stability and polarizability, high infrared transparency, and unique covalent structure of glasses containing CdO make them promising for use as photosensitive materials and in optoelectronic devices^13–15^. As a modifier, CdO causes many important structural modifications, including the concentration-dependent conversion of BO_3_ to BO_4_ units. Adding three-dimensional transition metal (TM) ions as dopants to glasses results in remarkable electrical, magnetic, and optical characteristics.

Unlike other TMs, vanadium is a strong ion that can be used in a variety of devices, including optical fibres, solar cells, solid-state lasers, and luminescence^16^. When using the traditional quenching procedure to make glass with the addition of additional ingredients, vanadium is necessary. Vanadium’s function, however, is contingent upon its concentration^17^. Vanadium may be seen as a former glass at elevated quantities and as a modifier at diminished amounts. The phase shifts induced by vanadium affect the optical, structural, and electrical characteristics of borate glass^18^. Recognized for its distinct pentavalent (V^5+^), tetravalent (V^4+^), and trivalent (V^3+^) states, vanadium exhibits characteristic absorption bands in glass matrices. Moreover, the ability of multivalent oxides to boost electrochemical activity and provide high conductivity makes mixed-valence vanadium oxide more appealing^19^. The type, content, and melting conditions of the glass all affect how often each valence state is^20^. To address the subtleties of vanadium’s behavior in glass, several glasses made of cadmium lead borate tellurite doped with vanadium ions were created and thoroughly examined^21^. V_2_O_5_ joins the glass network in the glass together with VO_5_ pyramidal structure units that contain V^4+^ and V^5+^ ions^22,23^. Vanadate glasses can have electrical conductivity as high as 10^−3^–10^−5^ Sm^−1^ because of the modest polaron hopping of 3 d^1^ unpaired electrons between V^4+^ and V^5+^ ions that exist in their structure^24,25^. Consequently, vanadate glasses are used in a variety of applications, including memory, optoelectronics, energy storage, photocatalysis and switching devices.

It is worth noting that there are previous studies on different borate glasses, and the effect of cadmium on properties is being studied, such as the effects of adding cadmium to bismuth borate glass^26^, nickel borate glass^15^, and iron lead borate^27^. However, the novelty in this manuscript is the preparation of a new system, which is cadmium borate, and small percentages of V_2_O_5_ up to 5 mol % were incorporated into it to study its effect on the structural, optical, mechanical, and dielectric properties.

## Experimental procedures

### Preparation of the glass samples

**Fig. 1:**
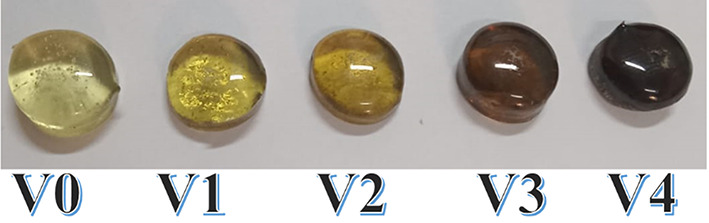
Photographs of prepared glass.

The experimental synthesis of homogeneous glasses involved utilizing high-purity powder reagents, specifically cadmium carbonate (CdCO_3_) with purity 99.95 for CdO was purchased from Spectrum Chemicals MFG, U.S.A., orthoboric acid (H_3_BO_3_) with purity 99.9% for B_2_O_3_ was obtained from SDFCL, Mumbai, India, and vanadium oxide (V_2_O_5_) with purity 99.99% purchased from Sigma Aldrich. Details of these materials’ composition are mentioned in Table 1. After being carefully measured and mixed into batches, these materials were melted at a high temperature. In a silicon carbide (SiC) heated furnace (produced by Vecstar, UK), this process was carried out in covered porcelain crucibles. The furnace was kept at a temperature of 1300 °C more than 90 min. The molten mixture was periodically stirred to obtain a homogenous molten. The homogenized melt was poured into a pre-warmed stainless-steel mold designed with specific dimensions (Fig. 1). As soon as the glass samples were cast, they were moved into an annealing muffle that had been pre-heated to a constant 400 °C. One hour later, the muffle’s power was cut off, allowing it to cool with a regulated rate of 25 °C per hour.

**Table 1:** Code and chemical composition of the prepared glass samples.

Sample code	Composition (mol%)		
	CdO	B_2_O_3_	V_2_O_5_
V0	70	30	0
V1	69	30	1
V2	68	30	2
V3	67	30	3
V4	65	30	5

### Infrared absorption spectra investigation

Fourier transform infrared (FTIR) spectroscopy (Bruker VERTEX 80 (Germany) combined Platinum Diamond ATR unit), which has a diamond disc as an internal reflector in the range 4000–400 cm^−1^ with resolution 4 cm^−1^ was used to measure FTIR absorption spectra to structurally characterize the building units of the prepared glasses.

### Density and molar volume

The glass sample was freely weighed in air and then submerged in xylene using the following relationship to get experimental density (ρ) measurements using the Archimedes principle:

1 $$\uprho =\frac{{\text{W}}_{a}}{{\text{W}}_{a}-{\text{W}}_{b}} {\uprho }_{b}$$

where ${\text{W}}_{a}$ is the weight in the air; ${\text{W}}_{b}$ is the weight of xylene and ${\uprho }_{b}$ is the density of xylene (${\uprho }_{b}$ = 0.865 g/cm^3^). The molar volume (Vm) can be calculated by dividing the molar mass (M) by mass density (ρ).

### UV–VIS spectroscopy

A UV–visible spectrophotometer (JASCO, FLH740, Japan) was used to measure the optical absorbance of the glasses in the 200–2400 nm range at a scan rate of 1000 nm/min using a D2/WI light source. The optical measurements were applied on polished glass samples of equal thickness (3 ± 0.1 mm).

### Mechanical properties

The Vickers microhardness (Hv) of polished samples was assessed using a Shimadzu-HMV (Japan) microhardness tester, applying a 100 g force under ambient laboratory conditions with a consistent indenter dwell period of 10 s. The microhardness was determined using the following equation^28^:

2 $$\text{Hv}= 1.854\frac{\text{ P}}{{\text{D}}^{2}}$$

P is the load in and D is the length of the diagonal. The fracture toughness, K_IC_, of the samples, was determined using indentation fracture utilizing a Vickers microhardness tester. K_IC_ was determined using this equation equation^29^.

3 $${\text{K}}_{\text{IC}}= 0.016 \frac{\text{Hv }{\text{a}}^{2}}{{\text{C}}^\frac{3}{2}}$$

where a is the half-length of the diagonal of the indent and c is the crack length from the center of the indentation to the crack end.

The longitudinal (V_L_) and shear (V_S_) velocities were measured using the pulse-echo technique. The Lame’s constants (i.e. λ and μ) were calculated using the bulk density of the sintered specimens according to the formula^30^:

4 $$\uplambda =\uprho ({V}_{L}^{2}-2{\text{V}}_{S}^{2})$$

5 $$\upmu =\uprho {V}_{S}^{2}$$

The longitudinal modulus, Young’s modulus, shear modulus, bulk modulus, and Poisson’s ratio (i.e. L, E, G, B, and υ respectively) of the sintered specimens were calculated according to the formula^31,32^:

6 $$L=\lambda +2\mu$$

7 $$E=\mu \frac{3\lambda +2\mu }{\lambda +\mu }$$

8 $$G=\mu$$

9 $$B=\lambda +\frac{2}{3}\mu$$

10 $$\upsilon = \frac{\lambda }{{2\left( {\lambda + \mu } \right)}}$$

### Dielectric properties

A broadband dielectric spectroscopy approach was used to evaluate the AC electrical conductivity, dielectric constant, and dielectric loss of the generated samples at room temperature.

The density, microhardness, fracture toughness, ultrasonic velocity, and a group of elastic moduli were measured four times, and the average was taken.

## Results and discussions

### Interpretation of the XRD, mapping, and FTIR spectra of the prepared glasses

**Fig. 2:**
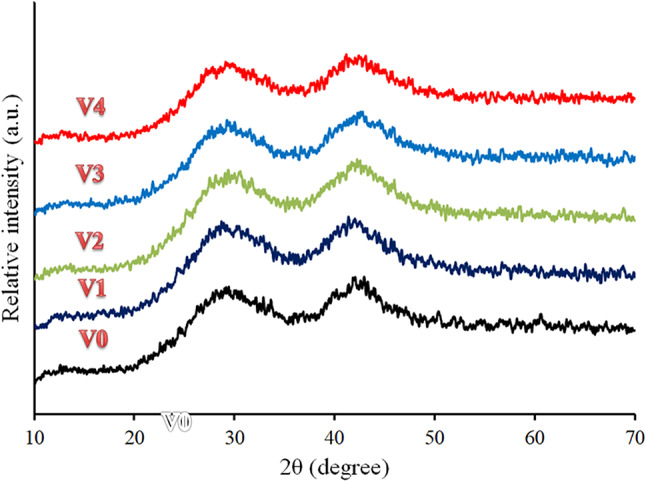
XRD patterns of all glass samples.

**Fig. 3:**
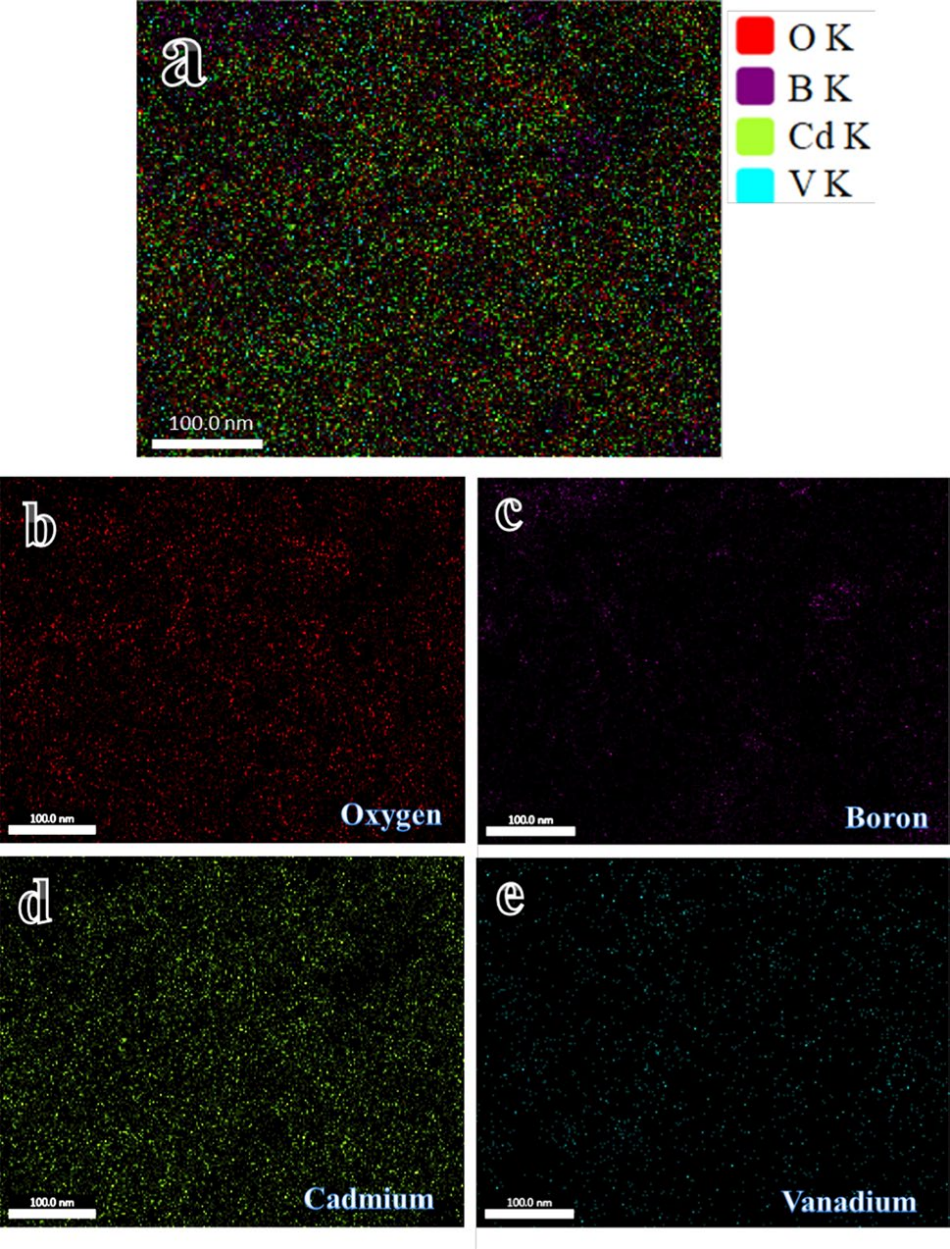
(**a**) EDX mapping of all constituents in the V4 sample, and the EDX mapping of the distribution of each component, i.e., (**b**) O, (**c**) B, (**d**) Cd, and (**e**) V.

Figure 2 illustrates the X-ray diffraction spectra of the produced glass samples. XRD analysis established the amorphous nature of these glasses. The spectrum displays only humps and does not show any sharp peak, indicating the amorphous nature of prepared glass samples. Figure 3a–e displays the EDX mapping of all the components that make up the V4 sample and the distribution of each element in it, i.e., O, B, Cd, and V. As can be seen, there is a good distribution of element components of glasses.

**Fig. 4:**
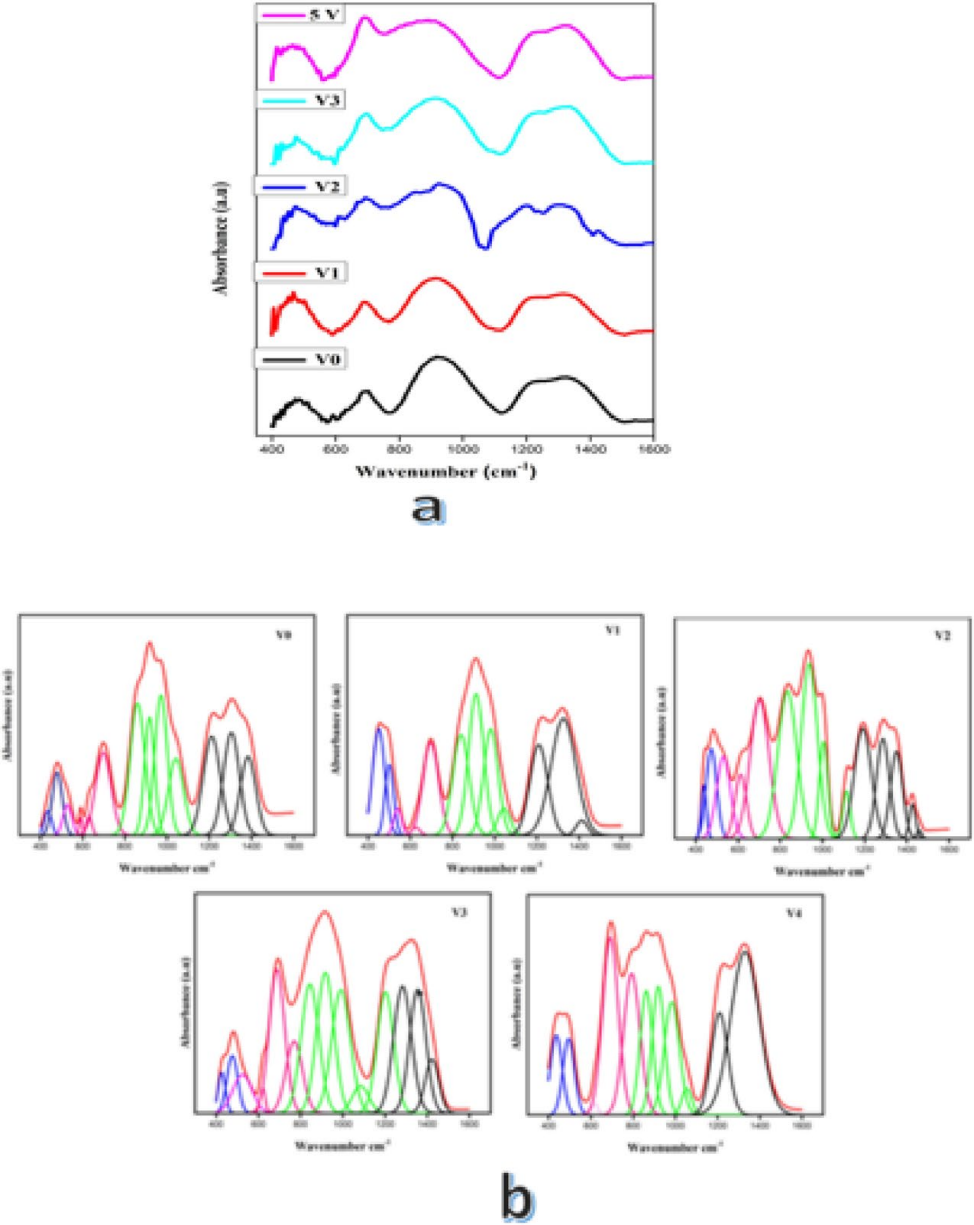
(**a**) FTIR spectra of the prepared glass samples and (**b**) their related deconvolution process.

The obtained spectra represented in Fig. 4 show various bands of the prepared glass samples in different spectral ranges extending from 400 to 1600 cm^−1^ with slight shifts in both band intensity and position. The addition of alkali, alkaline earth oxides, or some other metal oxides to borate glass enhances the transformation of some basic triangular (BO_3_) units to tetrahedral (BO_4_) units. This conversion continues until a certain limit, then the added oxide may cause a back conversion^33^. The vibrations of triangular borate units (BO_3_) are active in the wavenumber range 1200–1600 cm^−1^. In contrast, the lower frequency absorption in the range 800–1200 cm^−1^ belongs to the vibrational modes of tetrahedral borate units (BO_4_). The frequency of the mid-region (500–800 cm^−1^) is associated with the bending vibrations and deformation of various borate glasses. The weak bands detected in the region from 420 to 500 cm^−1^ are linked to V–O and Cd–O bonds in the glass network^34–37^. The broad bands observed in the regions from 800–1200 and 1200–1600 consist of several overlapped bands. So the deconvolution process is required to confirm each band separately. The infrared absorption region [800–1200 cm^−1^] is deconvoluted to four bands at 859, 917, 971, and 1042 cm^−1^, which are mainly identified as the B–O stretching vibrations of tetrahedral borate BO_4_ groups. The infrared absorption region [1200–1600 cm^−1^] is deconvoluted to three bands at 1211, 1305, and 1383 cm^−1^, and they are usually identified as the B–O stretching vibrations of triangular borate BO_3_ groups^12,38–41^.

The bands associated with the borate network were utilized to analyze the infrared spectra generated by the deconvolution process. The N_4_ ratio was among the characteristics that could be determined using the computed area under deconvoluted peaks. The following equation can be used to determine the N_4_ parameter, which gives important information on the BO_4_ ratio^38^:

11 $${\text{N}}_{4}=\frac{\text{The area of B}{\text{O}}_{4}}{\text{The total area of }({\text{BO}}_{3}+\text{ B}{\text{O}}_{4})}$$

**Fig. 5:**
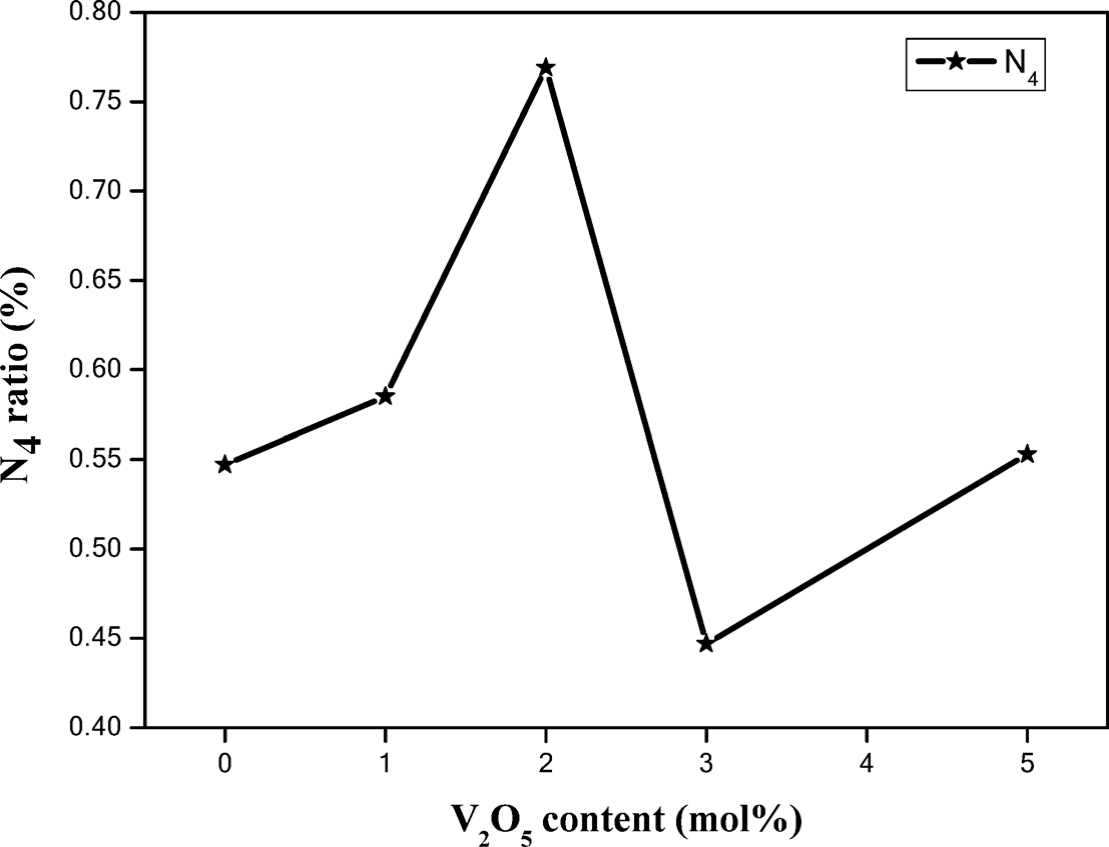
The dependence of *N*_4_ ratio on V_2_O_5_ content.

Figure 5 illustrates the irregular change in the N_4_ ratio as V_2_O_5_ content increases. Firstly, an increase is observed till a certain limit (2 mol%) and then the N_4_ value decreases in the 3 mol% and then shows a slight increase in the 5 mol% sample but is still lower than the 2 mol% sample. We can assert that the observed increase is attributed to the expected conversion of BO_3_ to BO_4_ by adding V_2_O_5_ to 2 mol%. The decrease that occurred at higher concentrations is due to the back conversion of BO_4_ to BO_3_ units. This assertion is supported by calculating the integrated area under peaks obtained from the deconvolution process as shown in Fig. 4.

### Density and molar volume

**Fig. 6:**
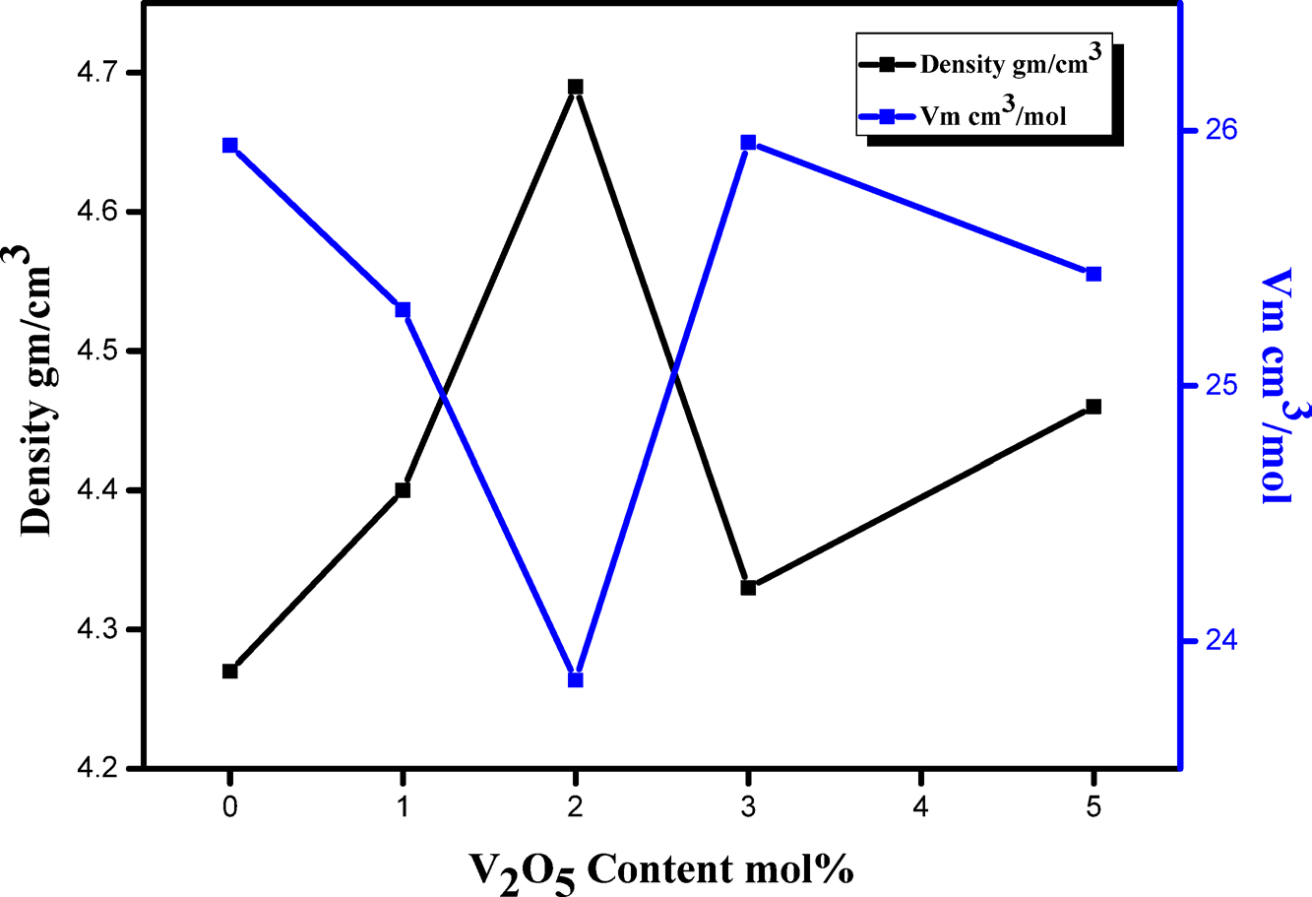
The relation between the density and molar volume of the glass samples as a function of V_2_O_5_ content.

The relationship between the density, ρ, and the molar volume, Vm, of the prepared glass samples as a function of V_2_O_5_ content is illustrated in Fig. 6. The values of ρ and Vm for the prepared glass compositions are recorded in Table 2. In general, introducing V_2_O_5_ to the glass network at the expense of CdO caused a noticeable change in the coordination number and dimension of interstitial spaces in the glass, causing a change in density. The density initially increases up to 2 mol%, which recorded the highest value, then decreases. The decrease in density is irregular, as the 5 mol% has a higher value than the 3 mol%, but both of them are lower than the 2 mol%. Similar behavior was reported for V_2_O_5_-doped borate glass^42^. The abovementioned data can be explained based on the previously calculated N_4_ ratio, which indicates that the 2 mol% sample has the highest N_4_ value (i.e., conversion of BO_3_ to BO_4_, which by role enhances the formation of the V_2_O_5_ phase rather than the CdO_4_ phase). As a result of enhancing the formation of V_2_O_5_, the network structure becomes more compact due to the small atomic radius of vanadium compared to cadmium. In the case of concentrations higher than 2 mol%, the N_4_ ratio decreases, and hence the network structure may become more disordered or less tightly packed, leading to a reduction in density^43,44^. Furthermore, in the prepared glass samples, Vm shows the same trend that occurred in the density, but in an opposite direction. The molar volume decreases gradually up to 2 mol%, which recorded the lowest value, then increases. The increase in molar volume values beyond the 2 mol% sample is also irregular, as the same occurred in density. The explanation for the behavior occurring in the Vm is also due to the N_4_ ratio.

**Table 2:** Density (*ρ*) and molar volume (Vm) for the prepared samples.

Sample	Density (*ρ*)	Vm	M_wt_
V0	4.27	25.94145	110.77
V1	4.4	25.2977	111.31
V2	4.69	23.8465	111.84
V3	4.33	25.9538	112.38
V4	4.46	25.437	113.45

### UV–visible absorption spectra

Vanadium’s UV–Vis characteristics in glass structures provide important information about the structural functions and optical behavior of vanadium ions. V_2_O_5_ alters the optical absorption properties of different glass matrices by affecting the electronic structure and the connectivity of the glass network^45,46^. As V_2_O_5_ is present, the absorption edge gradually shifts towards higher wavelengths, signifying a rise in the concentration of vanadyl species^47,48^.

**Fig. 7:**
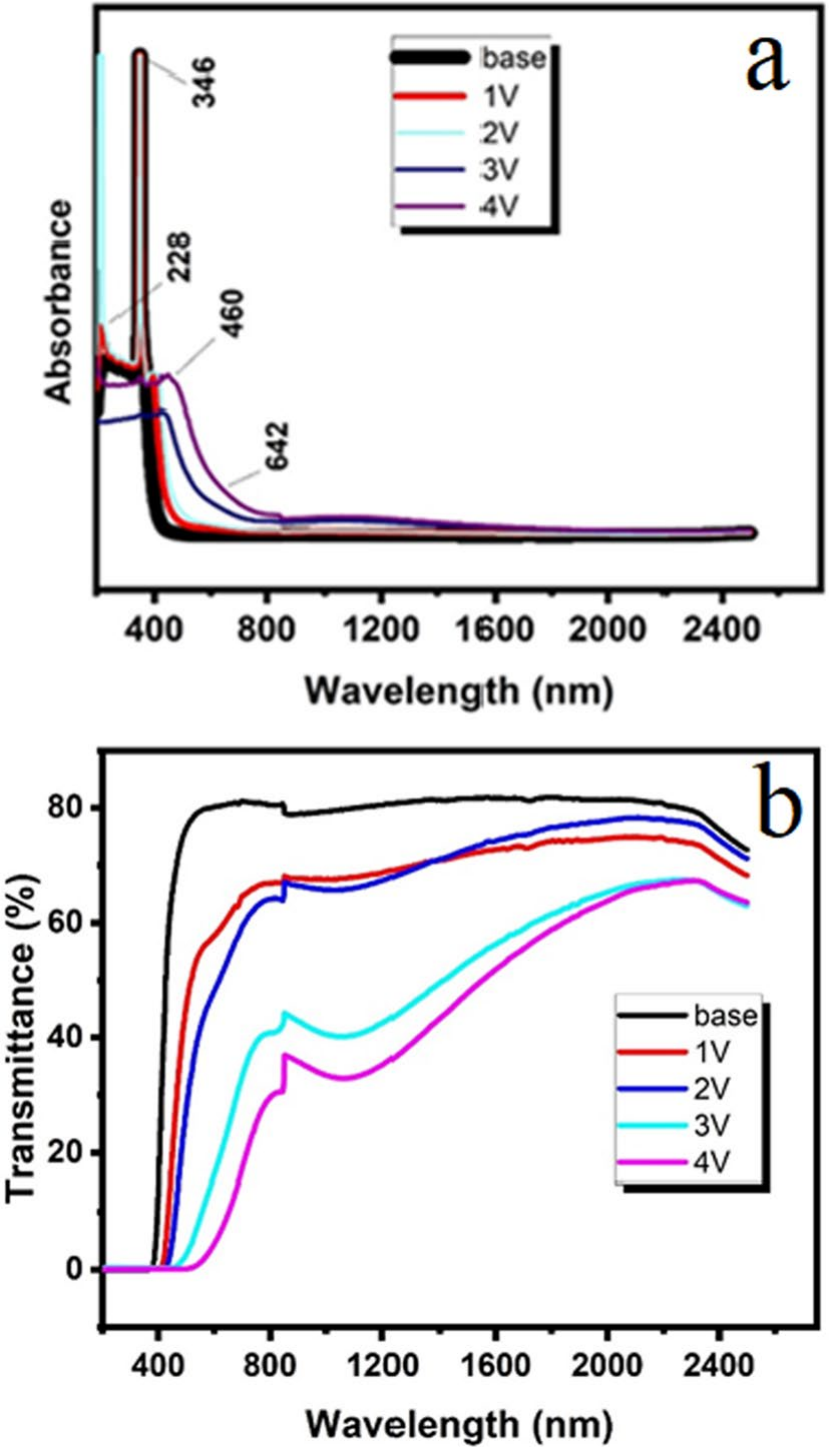
(**a**) Optical absorption spectra, and (**b**) optical transmittance spectra of the prepared glass samples.

Figure 7 shows the absorption spectra for (30 B_2_O_3_. (70−x) CdO. x V_2_O_5_: x = 0, 1, 2, 3, and 5 mol%). The absorption spectra contained four bands at 228, 346, 460, and 642 nm. As seen in Fig. 5a,b, the addition of V_2_O_5_ resulted in a decrease in transmittance (T) and an increase in absorbance (A). As density increases, variations in optical parameters (A and T) can be explained^46,49^. Because of the multi-oxidation states in V_2_O_5_, the optical band gap, E_g_, reduced from 2.95 to 1.35 eV as the V_2_O_5_ content increased. This suggests that there are more localized states in the glass matrix, which are associated with higher absorbance and lower transmittance^50^. For the examined glasses, the absorption coefficient (∝) was calculated as follows:

12 $$\propto = \left( {2.303/{\text{d}}} \right){\text{ A}}$$

where A is the absorbance and *d* is the thickness of the glasses^51^.

**Fig. 8:**
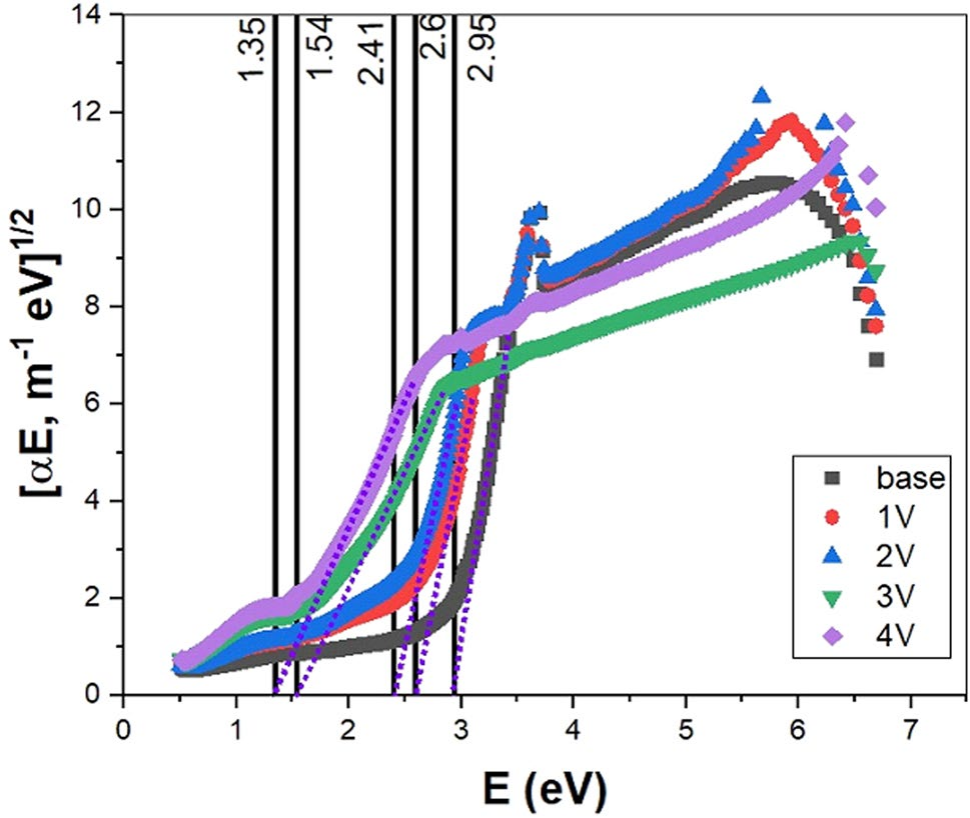
The optical band gap for indirect electronic transitions.

All glass samples’ optical energy gaps (Eg) have been determined using the Davis–Mott relation, which is equivalent to Ref.^52^. The authors used Taucn’s relation to determine the optical band gap for indirect electronic transitions, as illustrated in Fig. 8. The obtained results validate that the addition of V_2_O_5_ alters the electrical structure of glass by decreasing the band gap’s width.

### Mechanical properties

**Fig. 9:**
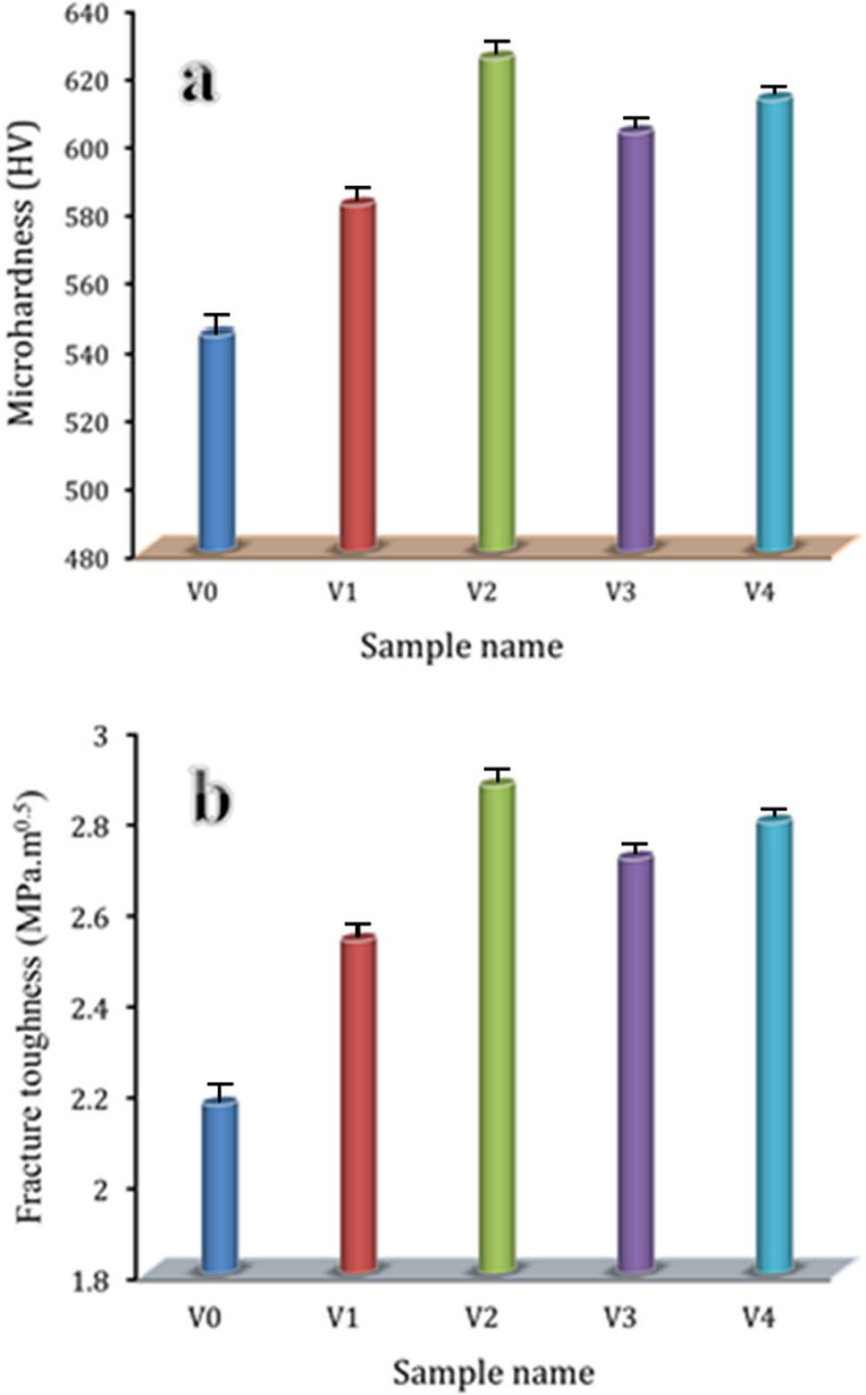
Effect of V_2_O_5_ mol%. on microhardness and fracture toughness of cadmium borate glass.

Microstructure and phase crystalline assembly have an impact on the mechanical properties of glass. Resistance to abrasion and scratching is a frequent interpretation of glass hardness. Glass characteristics vary greatly depending on its chemical makeup^53–55^. The effect addition of V_2_O_5_ mol% on Vicker microhardness and, fracture toughness of cadmium borate glass is displayed in Fig. 9. The figure shows that an increase in V_2_O_5_ contents up to 2 mol% is associated with a noticeable increase in microhardness and fracture toughness, but adding 3 mol% of V_2_O_5_ causes a decrease in both, then they increase again by increasing the V_2_O_5_ to 5 mol%. The microhardness and fracture toughness of undoped cadmium borate glass (V0) are 543.12 HV and 2.17 MPa.m^0.5^, respectively. It found that When compared with the previous glass sample, the microhardness, after doped 1 mol% V_2_O_5_ (V1), the microhardness and fracture toughness, increased to 581,04 HV and 2.53 MPa.m^0.5^ which improved by about 6.89, and 16.59%, respectively. The sample doped 2 mol% V_2_O_5_ (V2) contains an increase in microhardness and fracture roughness record 624.12 HV and 2.87 MPa.m^0.5^ which improved about 14.91, and 32.26%. As the V_2_O_5_ continues to increase (V3), it results in a decrease in both microhardness and fracture toughness recorded 602.23 HV and 2.70 MPa which decrease about 3.51, and 5.51%, respectively, compared to the V2 sample. Slight increase in microhardness and fracture toughness after doped 5 mol% V_2_O_5_ (V4) which records 611.97 HV and 2.79 which increases about 1.62, and 3.3% compared to the V4 sample.

**Fig. 10:**
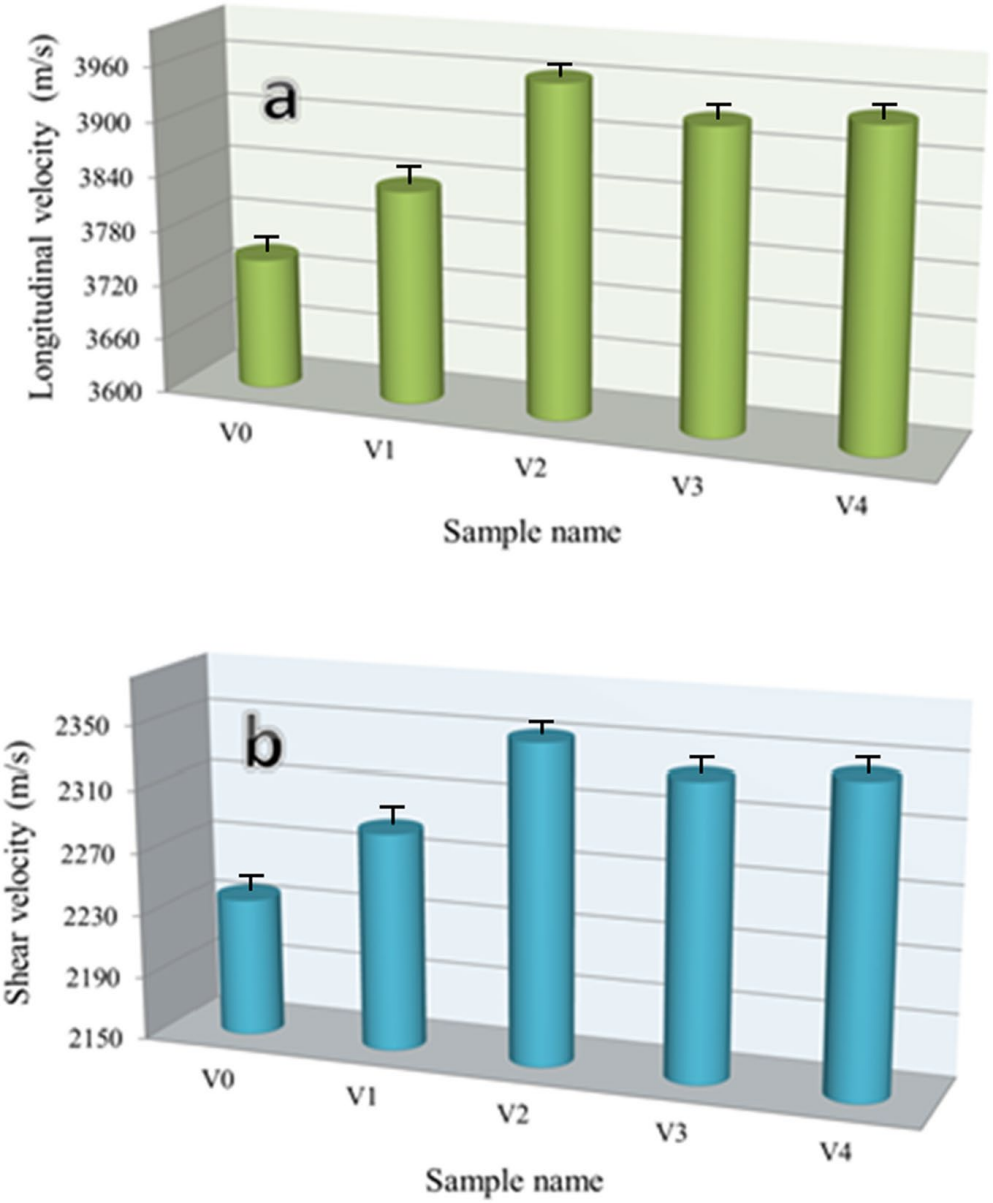
Ultrasonic velocity of cadmium borate glass with V_2_O_5_ mol%.

**Fig. 11:**
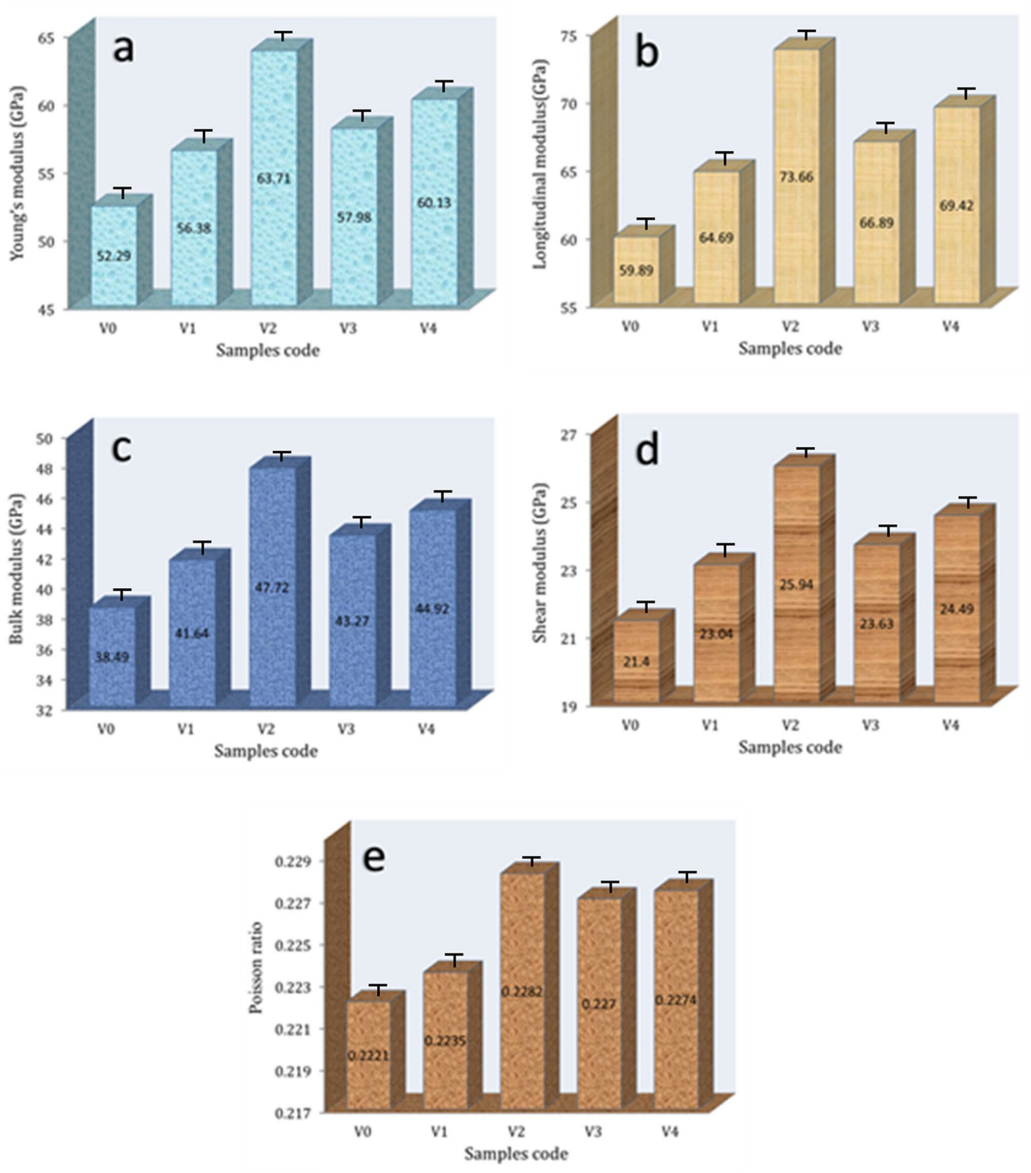
Elastic moduli of V0, V1, V2, V3, and V4 glass samples.

The ultrasonic velocities and the group of elastic moduli for all glass samples were measured and shown in Figs. 10 and 11, respectively. As clearly shown by these figures, ultrasonic velocities and group elastic moduli show the same trend of microhardness and fracture toughness, that is, as discussed former, where they exhibit significant increases in their values with the added 1 and 2 mol% V_2_O_5_ to cadmium borate glass. By adding 3 mol% V_2_O_5_, the values ​​of ultrasonic velocity and elasticity moduli decrease compared to the previous sample, while adding 5 mol% V_2_O_5_ increases again. The longitudinal velocities of V0, V1, V2, V3, and V4 are 3744.21, 3834.28, 3962.54, 3930.08, and 3944.07 m/s, respectively and shear velocities are 2237.90, 2288.51,2351.39, 2335.59, 2342.98 m/s, respectively. Moreover, the Young’s modulus for the same samples are 52.29, 56.38, 63.71, 57.98, and 60.13, respectively. There is no doubt that the change in glass density has a clear effect on the mechanical properties such as microhardness, elastic moduli, and fracture toughness, as increasing the density leads to an improvement in the mechanical properties of the glass and vice versa^56,57^. The addition of V_2_O_5_ initially results in an improvement in the mechanical properties of the glass up to 2 mol%. This is because the V_2_O_5_ pentoxide serves as a network modifier, therefore adding stronger V–O linkages into the structure of the glass. Additionally, it forms cross-links which include V–O–V and B–O–V, which strengthen the structural rigidity and increase the the mechanical properties of the glass. On the other hand, for sample V3, the network is weakened because of the loss in mechanical properties that occurs as a result of the addition of V_2_O_5_. This is because the excessive introduction of non-bridging oxygens (NBOs) causes the network to have less strong cross-links. Which configuration of non-bonding orbitals breaks the glass network by severing an excessive number of B–O–B links, resulting in a more open and less robust structure. Through the formation of extra V–O bonds or the rearrangement of the glass matrix, excessive V_2_O_5_ can reintroduce stronger cross-links, hence increasing the structural mechanical properties of the glass (V4)^58–60^.

### Dielectric properties

By maintaining the molar ratio of the lattice (B_2_O_3_) constant while increasing V_2_O_5_ at the cost of CdO for the lattice-which is regarded as ionic inactive because of the poor mobility of Cd^+2^ ions inside the lattice-the electrical characteristics of the glass samples were examined. At room temperature and throughout a frequency range of 1 Hz to 20 MHz, the electrical characteristics of the produced glasses, including their AC electrical conductivity, dielectric constant, and dielectric loss, were examined.

**Fig. 12:**
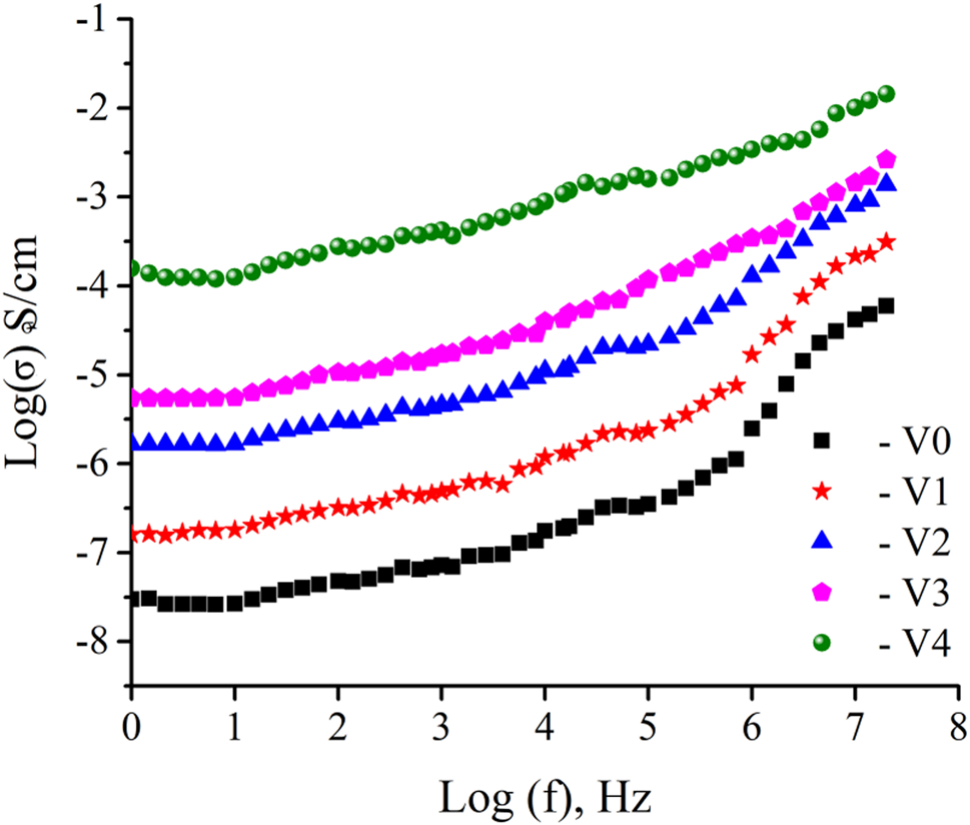
The ac-conductivity of cadmium borate glass doped with 0, 1, 2, 3, 5 mol% V_2_O_5_ at various temperatures.

The frequency dependence of the electrical conductivity, σ (w), for all glass samples, is displayed in Fig. 12. As can be seen from the image, conductivity is frequency-independent at low frequencies ) ≤ 10^3^ Hz) and this characteristic of this relationship is a plateau area corresponding with the dc-conductivity. On the other hand, at high frequencies (≥ 10^6^ Hz), there is a change in slope as a consequence of increasing conductivity. For example at low frequency (10 Hz), the electrical conductivity of V0, V1, V2, V3, and V4 are 2.68 × 10^–8^, 1.81 × 10^–7^, 1.68 × 10^–6^, 5.53 × 10^–6^, and 1.62 × 10^–4^, respectively while at high frequency (20 MHz), the conductivity of same previous samples are 5.96 × 10^–5^, 3.11 × 10^–4^, 5.53 × 10^–3^, 2.62 × 10^–2^, and 1.44 × 10^–2^ respectively. This is something that can be noticed considering that conductivity has shifted from being frequency-independent to being frequency-dependent, a phenomenon known as conductivity relaxation may have been established^61–63^. Increasing in conductivity has been seen in an area that is characterized by high frequencies. The σ (f) values for all glass samples conform to the general relation dictated by Jonscher’s universal lower law^56,64^:

13 $$\sigma \left(f\right)= \sigma \left(0\right)+A {f}^{S}$$

where σ (0) is dc conductivity (the conductivity at zero frequency), A is a constant, and S is the frequency exponent. It is known that the exponent S denotes the degree of interaction among mobile ions and surrounding lattices^65^. It is worth noting that a CdO-B_2_O_3_ glass system is generally a semiconductor material with a structure composed of 70 mol% CdO is a semiconductor and 30 mol% B_2_O_3_ is an insulator. This is due to CdO imparting a certain level of electronic conductivity, but B₂O₃, functioning as a network former, generates a comparatively stable glass matrix that restricts carrier mobility.

The vitreous composition depends on a significant amount of CdO, which interferes with the borate network to become disorganized by causing part of the B-O-B bridges to be broken and resulting in the formation of non-bridging oxygens (NBOs). These NBOs may provide local venues that facilitate ion mobility or limited electronic delivery. On the other hand, cadmium ions (Cd^2^⁺) do not contribute to the movement of free electrons in the way that a metal might do, while conduction is limited to movement between local states or between cadmium ions, which contributes to the nature of semiconductors.

It is worth noting that there are two distinct processes in improved electrical conductivity. Firstly, by hopping a mobile electron from a low- to a high-valance state, doping transition metal ions in cadmium borate glasses improves their electrical conductivity (electronic conduction)^66^. As a result, the addition of V ions increases the ac-conductivity by establishing pathways for the passage of the charge carriers (Cd ions). The second is ionic conduction because of how cadmium ions move. However, since more V_2_O_5_ was present, more V^4+^ ions were converted to V^5+^ ions, increasing electronic conduction. The conductivity data show that V^5+^ is present and supports the IR spectra. The NBOs may identify the conducting channel, enabling Cd^2^⁺ ions to be disrupted via the glass matrix and by electrical transmission in another manner^60,67^. It is also noticeable from the figure that the effect of 3 mol% V_2_O_5_ (V3) is not significant compared to sample V2 and has a close relationship with the decrease in density.

**Fig. 13:**
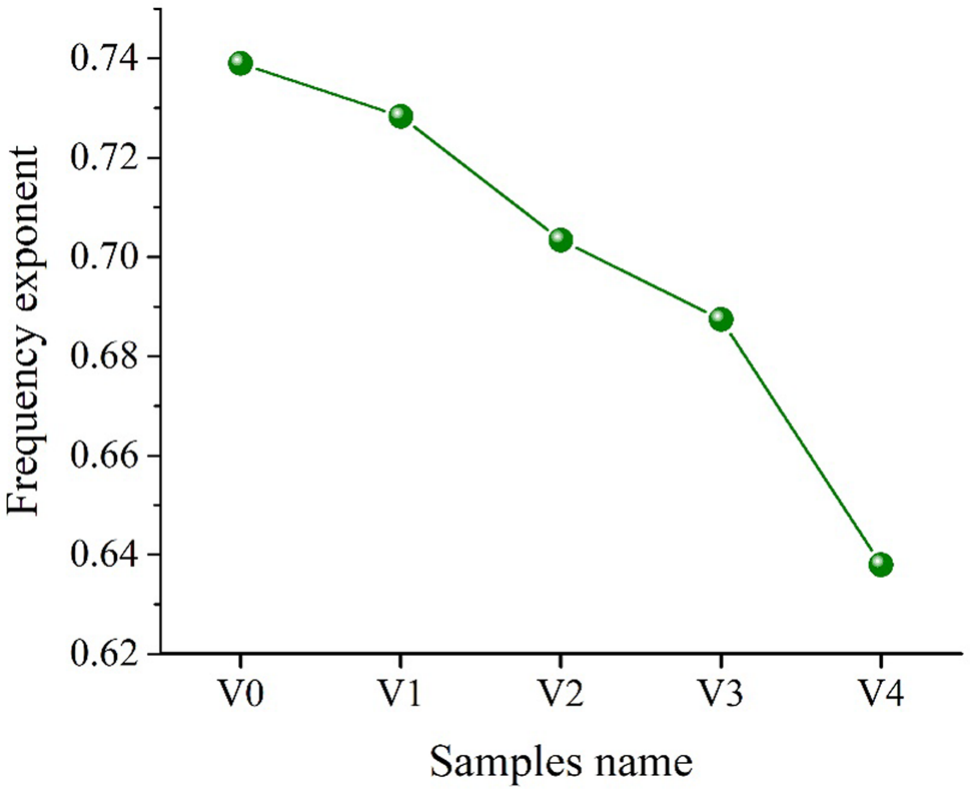
Variation of frequency exponent with doped V_2_O_4_ for cadmium borate glass.

Figure 13 displays the effect of mol% of V_2_O_5_ on the variation of frequency exponent (S) values of glass samples. This decrease in the value of S is mainly due to the creation of NBO atoms, which interfere with the glass network structure^68^. There is a possibility that the decreased exponent seen for V1, V2, V3, and V4 glass samples compared to those observed in cadmium borate glasses (V0) is due to increased polaronic conduction and enhanced charge carrier hopping mechanisms.

Dielectric materials are defined by their capacity to retain energy when subjected to an external electric field. In every instance, there exists an induced polarization resulting from the interaction between the external electric field and the field generated by the examined sample. Generally, the complex permittivity, which is composed of real and imaginary components, is used to characterize the behavior of dielectric materials^57,69^.

14 $$\varepsilon^{*} = \varepsilon^{\prime } - i\varepsilon^{\prime \prime }$$

where ε* is the complex dielectric permittivity, and ε′, and ε″ are the real and imaginary parts of the dielectric constant, respectively, and i is a constant (imaginary unit). The imaginary part estimates the energy lost as a result of frictional damping that prevents the displacement of charges from being in phase with the field change, while the real part measures the energy stored in the dielectric material as a response to the application of an electric field. These are determined using the following expressions^70,71^:

15 $$\upvarepsilon ^{\prime } = \frac{{{\text{Cd}}}}{{{\text{A}} \in _{0} }}$$

16 $$\varepsilon^{\prime \prime } = \varepsilon^{\prime } \tan \delta$$

where A is the sample’s cross-sectional area in square meters, d is the glass sample’s thickness in meters, ε_0_ is the permittivity of free space (ε_0_ = 8.85 × 10^−12^ F/m), and (C) is the sample capacitance and tan δ = the tangent loss. The term tan δ provides the phase difference resulting from the energy loss inside the structure based on the values of ε′ and ε″. This indicates that the electrical loss is represented by the dissipation factor tan δ. The ratio of electrical energy wasted in heat to the total electrical energy stored every cycle in the dielectric materials is known as the dissipation factor or tan δ.

**Fig. 14:**
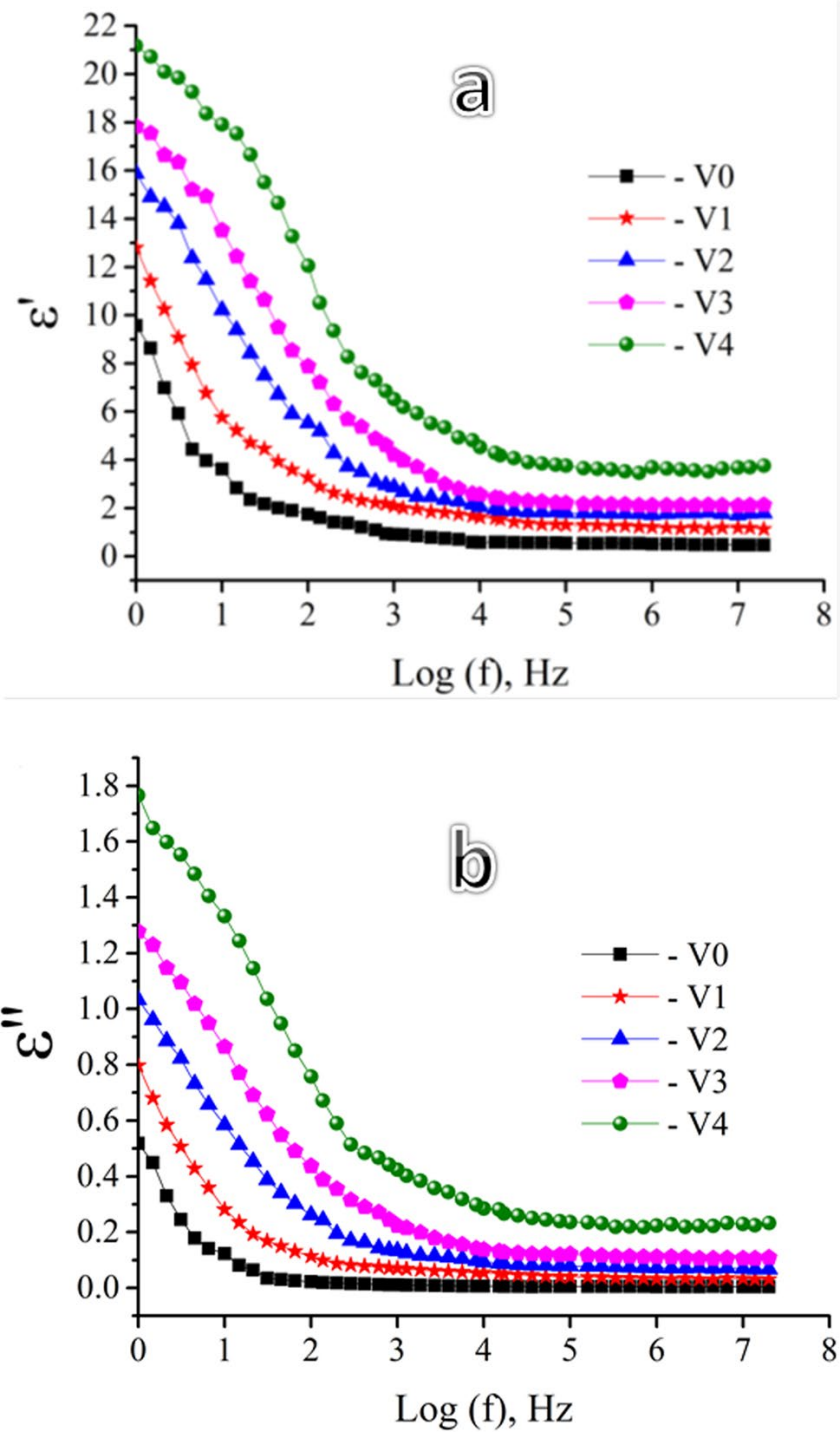
Real and Imaginary parts of the dielectric constant versus frequency plot for all sample glass at various temperatures.

Figure 14 shows the frequency dependence of the real and imaginary dielectric constant with and without mol percentage of V_2_O_5_ at room temperature. It is clear from the figure that adding V_2_O_5_ has a positive effect on the dielectric increase, especially at low frequencies. The reason for this rise in the real and imaginary dielectric constant of cadmium borate glass with the addition of V_2_O_5_ is that V_2_O_5_ serves as a network modifier, introducing NBOs that improve the mobility of charge carriers. There is an increase in the dielectric constant as a result of these NBOs since they permit greater dipole moments^60,72^. Moreover, the curves represented exhibit a rapid reduction in ε′ and ε″ as the frequency increases. However, after the frequency reaches 100 kHz, the pace of the decrease in ε slows down and becomes mostly constant. First, the total polarization which is the sum of all polarization types, including electronic, ionic, dipolar, and space charge is responsible for the dielectric constant. A greater dielectric constant may result from several polarization processes that follow the applied electric field at low frequencies^73^. The slower polarization processes, such as dipolar and interfacial polarization, are unable to keep up with the electric field’s fast oscillations as frequency rises, which decreases their contribution to ε′ and ε′′. Only electronic polarization, which contributes much less than the slower processes, is eventually still active at extremely high frequencies^74^. Charge carriers enhance ε′ and ε′′ at low frequencies by accumulating at structural inhomogeneities, such as grain boundaries or phase-separated areas. Interfacial polarization stops playing a role at higher frequencies because charge carriers are unable to keep up with the quickly shifting field^75,76^.

**Fig. 15:**
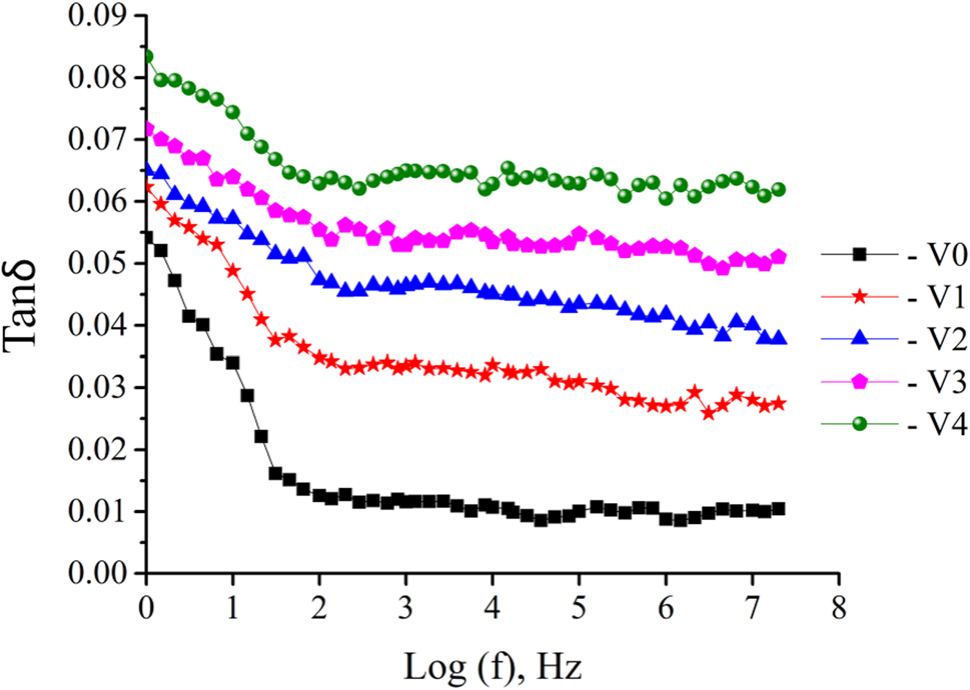
Variation of tan δ for various glasses samples with the applied frequency at.

At room temperature, the change of the dielectric loss tangent (tan δ) with frequency is shown in Fig. 15. This variation is seen for glasses that contain 0, 1, 2, 3, and 5 mol% of V_2_O_5_. It is evident from this figure that the changes in loss tangent are very much comparable to those of the real and imaginary dielectric constant. This is something that can be determined with absolute certainty. The hopping frequency may be unable to synchronize with the applied frequency at higher frequencies, which results in a reduction in the frequency dependency. This may be the reason why the dielectric loss diminishes as the frequency increases and becomes nearly frequency-independent at higher frequencies^77,78^.

## Conclusion

Samples of cadmium borate glass were synthesized by substituting V_2_O_5_ for CdO. FTIR studies confirmed the effective role of V_2_O_5_ on the structural vibrations of the functional groups in the glass matrix, as the increase in V_2_O_5_ concentration made a noticeable change in the N_4_ ratio, confirming the conversion of BO_3_ to BO_4_ till a certain limit (2 mol %), then the back conversion of BO_4_ to BO_3_ units occurred at higher concentrations. Increasing the V_2_O_5_ phase at the expense of the CdO_4_ phase enhanced the network structure to become more compact due to the small atomic radius of vanadium compared to cadmium. As a result, the density initially increased on adding V_2_O_5_ up to 2 mol% (the best concentration enhanced the formation of the V_2_O_5_ phase rather than the CdO_4_ phase) and then decreased. The molar volume followed the same trend that occurred in the density but in an opposite direction. UV–Vis characteristics validated that the addition of V_2_O_5_ resulted in shifting the absorption edge towards higher wavelengths. Moreover, the addition of vanadium has a positive effect on improving the mechanical properties of cadmium borate glass, although the mechanical properties of sample V3 decreased slightly compared to sample V2, then they increased again in sample V4. The conductivity is identified to increase with increasing both frequency and V_2_O_5_ addition, while the dielectric constant (real & imaginary) and tangent (tan δ) reduced with increased frequency and increased with the addition of V_2_O_5_ The S value of cadmium borate glass (V0) is 0.739, and after adding 1, 2, 3, and 5 V_2_O_5_, the values decrease to 0.728, 0.703, 0.687, and 0.638, respectively, due to the creation of NBO atoms.

## Data Availability

The datasets generated and/or analyzed during the current study are not publicly available because all data are presented in the article and therefore, there is no need to include raw data but they are available from the corresponding author upon reasonable request.
